# The Challenges and Hopes in Treating Patients with NOA

**DOI:** 10.3390/jcm12062191

**Published:** 2023-03-12

**Authors:** Shevach Friedler

**Affiliations:** 1 IVF Unit, Barzilai University Medical Center, Ashkelon 7830604, Israel; prof.friedler.s@gmail.com; 2 Faculty of Health Sciences, Ben-Gurion University of the Negev, P.O. Box 151, Beer Sheva 84101, Israel

Infertility due to the male factor occurs in no less than 50% of investigated couples. For 1% of these couples, sperm is absent in the ejaculate, representing the group of patients with the most frustrating problem. The reason for their azoospermia is, in the majority of cases (60–70%), related to some form of testicular failure to produce spermatozoa (non-obstructive azoospermia—NOA), whereas the remaining cases are due to some form of obstruction preventing the available sperm cells to reach the ejaculate (obstructive azoospermia—OA).

When I was a medical student several decades ago, I was taught that for patients diagnosed with azoospermia, the only solution is the use of donor sperm. This is not true anymore thanks to the invention of ICSI, which was first introduced in 1992 and then applied successfully using testicular sperm in 1993. In 1995, the surgical extraction of testicular sperm to be used for ICSI achieved livebirth and gave hope to patients with NOA.

The primary problem a clinician must solve in the case of a patient with azoospermia is to make a correct differential diagnosis and workup to differentiate between patients suffering from obstructive or non-obstructive azoospermia, as the prognosis, methods of intervention and outcome differ between them. Andrade et al. [1] present an excellent manuscript describing the current knowledge concerning the differential diagnosis between OA and NOA, describing the importance of thorough medical history collection, physical examination, routine sperm analysis, hormonal evaluation, imaging techniques and genetic analysis. The place for testicular biopsy has changed from a diagnostic procedure in the past to the current method of treatment, enabling the immediate use of the testicular spermatozoa for ICSI. Some interesting clinical cases presented by the authors offer the reader exposure to the clinical management of a variety of men with azoospermia, increasing their ability to deal with real-life situations faced when treating such couples. 

Confrontation with a patient diagnosed with azoospermia requires a thorough workup to estimate whether it is due to obstruction or non-obstructive azoospermia. Still, it is quite frustrating to not be able, in many cases, to offer a precise diagnosis for the patient that explains his specific situation. The etiology for NOA in different patients may be variable and genetic tests are constantly evolving to offer the possibility of diagnosing the specific genetic mishap that may explain the problem. The thorough narrative review by Krausz and Cioppi [2] offers the reader an excellent overview of the current genetic tests available, with a hint also toward the future. The classical examination of karyotype or Y deletions may be enriched by WES, which can also diagnose some monogenic causes for NOA. The diagnosis is important not only from the azoospermia perspective but also due to the general health risks involved for the patients and their potential children.

Kang et al. [3] present a comprehensive overview of the variable causes for NOA, discussing possible hormonal interventions to optimize either spontaneous sperm production or increase the surgical sperm retrieval rate, and describe variable surgical methods with their relative success rates and according to the variable etiologies for NOA such as Kleinfelter’s syndrome, Y deletions, previous chemotherapy, or cryptorchidism. The outcome of ICSI is influenced by etiology, sperm source and the laboratory technique used for sperm selection. Finally, the authors describe variable laboratory interventions that are being developed in the search to optimize clinical results. 

When confronting a patient with NOA, one has to accurately estimate the chances for finding testicular sperm following the surgical intervention. As clearly presented by Caroppo and Colpi [4], the predictive tools and parameters available to the clinician at the moment are not good enough to estimate accurately testicular sperm production and still warrant optimization in the clinical context. The various predictive models for successful sperm retrieval in patients diagnosed with NOA include clinical parameters such as their genetic background, presence of varicocele and history of cryptorchidism, testicular volume and hormonal parameters. T histology of the testicular tissue is interesting and related to the cause of spermatogenesis failure, but it is available to the clinician only after surgical intervention. Variable molecular markers in the seminal fluid, such as germ cell-specific mRNAs, extracellular vesicles containing variable microRNAs (miRNAs), and long non-coding RNAs are being examined to evaluate their potential in the prediction of whether sperm cells are present in the testicles, but none has proven to be efficient enough to be offered to the patients outside of research. As the sperm retrieval rate may be influenced by other factors, such as the surgeon’s competence, the sperm retrieval method used and the laboratory’s efficiency in terms of identifying the sperm cells, developing a correct, universal predictive model is challenging and open to further research.

Considering that the successful sperm retrieval rate in patients with NOA is about 50%, the optimization of the procedure is required. As normal spermatogenesis requires the presence of intratesticular testosterone and surgical sperm retrieval rates were observed to be lower in patients with lower testosterone levels, the optimization of its levels was offered as an adjunctive therapy to be applied before TESE. Caroppo and Colpi [5] present a challenging review, highlighting rational and possible medical interventions to increase testosterone levels in patients with NOA. After a very concise and clear explanation of the roles of both FSH and testosterone in spermatogenesis, they presented the available data in the literature, showing the correlation between success in sperm retrieval and presurgical testosterone and FSH levels. They underlined the fact that the literature lacks sufficient prospective data to reach a definitive conclusion regarding the indications and methods of treatment, although patients with normal testosterone levels had a better sperm retrieval rate compared with those with lower serum testosterone.

Punjani et al. [6] offer a nice narrative review of the history of the evolvement of the surgical sperm retrieval techniques, including fine-needle aspiration and multiple testicular biopsies—which led to the introduction of microsurgical testicular sperm extraction by Schlegel et al. in 1998–that became the gold-standard technique used today for patients with NOA. Unfortunately, none of the predictive factors suggested, including testicular volume, FSH level, age, cryptorchidism etc., have a good enough predictive value, although some genetic factors (such as AZF deletion, type a or b) may preclude spermatogenesis entirely. Whether or not micro-TESE is more efficient in terms of sperm retrieval rate than the conventional TESE is debatable due to the presence of several meta-analyzes in the literature reaching different results. Patients with NOA should be advised before the surgery to consider the need for special pre-surgical interventions such as varicocelectomy or hormonal therapy to increase intratesticular testosterone levels, aiming to optimize their sperm retrieval rate. Improving laboratory techniques for sperm cell identification or finding better tools to locate the foci with spermatogenesis in the testicles may help to improve the chances of successful sperm retrieval.

Colpi and Caroppo [7] share with readers a clear and concise review of microsurgical sperm extraction. They emphasize the need to consider pre-surgical optimization when needed, as well as the need to use accurate methodology in the performance of the procedure to optimize clinical outcomes. The detailed methodology of the surgical procedure is exceptionally well-described and accompanied by clear pictures taken from the operating microscope. The postoperative course is described, helping the reader to learn accurately and precisely about the performance of micro-TESE.

The surgical sperm retrieval procedure is not without any harm. As presented in the excellent review by Billa et al. [8], a follow up after conventional or micro-TESE is required as damage to the Leydig cells may cause a decrease in their functioning in the production of natural testosterone. The disturbance may be temporary or permanent, probably related to the etiology of NOA, the initial status of the testicles in terms of size and endocrine function, and the amount of damage caused by the surgical intervention, including the number of incisions, quantity of tissue extracted and the technique of the surgeon. The most vulnerable subgroup is made up of patients diagnosed with Klinefelter’s syndrome. This review emphasizes the need for patient follow up, as in most cases the temporary decline in testosterone may improve spontaneously after 12–18 months.

To conclude, the challenge of the andrologist to help a patient with NOA is quite remarkable. It requires using all the available tools to establish a correct diagnosis, decide upon the need for presurgical optimization to enhance spermatogenesis, and evaluate his prospects to find viable sperm in his testicles to be used for ICSI, choosing the most appropriate surgical intervention and most suitable laboratory technology. Or else, based on the current knowledge, they should counsel against surgical intervention when the prospects to find testicular sperm are nil and help the patient find alternative solutions to complete his family.
